# Experiences of caregivers of children with inherited metabolic diseases: a qualitative study

**DOI:** 10.1186/s13023-016-0548-2

**Published:** 2016-12-07

**Authors:** Shabnaz Siddiq, Brenda J. Wilson, Ian D. Graham, Monica Lamoureux, Sara D. Khangura, Kylie Tingley, Laure Tessier, Pranesh Chakraborty, Doug Coyle, Sarah Dyack, Jane Gillis, Cheryl Greenberg, Robin Z. Hayeems, Shailly Jain-Ghai, Jonathan B. Kronick, Anne-Marie Laberge, Julian Little, John J. Mitchell, Chitra Prasad, Komudi Siriwardena, Rebecca Sparkes, Kathy N. Speechley, Sylvia Stockler, Yannis Trakadis, Sarah Wafa, Jagdeep Walia, Kumanan Wilson, Nataliya Yuskiv, Beth K. Potter, on behalf of the Canadian Inherited Metabolic Diseases Research Network (CIMDRN)

**Affiliations:** 1 Faculty of Medicine, School of Epidemiology, Public Health and Preventive Medicine, University of Ottawa, 451 Smyth Road, Ottawa, ON K1H 8M5 Canada; 2 Ottawa Hospital Research Institute, Ottawa, ON Canada; 3 Newborn Screening Ontario, Children’s Hospital of Eastern Ontario (CHEO), Ottawa, ON Canada; 4 Department of Pediatrics, Dalhousie University and IWK Health Centre, Halifax, NS Canada; 5 University of British Columbia, BC Children’s Hospital, Vancouver, BC Canada; 6 Department of Pediatrics and Child Health, University of Manitoba and Children’s Hospital Research Institute of Manitoba, Winnipeg, MB Canada; 7 Institute of Health Policy Management and Evaluation, University of Toronto, Toronto, ON Canada; 8 Program in Child Health Evaluative Sciences, The Hospital for Sick Children, Toronto, ON Canada; 9 University of Alberta, Stollery Children’s Hospital, Edmonton, AB Canada; 10 Division of Clinical and Metabolic Genetics, University of Toronto and The Hospital for Sick Children, Toronto, ON Canada; 11 Medical Genetics, Department of Pediatrics, CHU Sainte-Justine, Montréal, QC Canada; 12 McGill University Health Centre, The Montreal Children’s Hospital, Montréal, QC Canada; 13 Genetics, Metabolism and Paediatrics, London Health Sciences Centre, Western University, London, ON Canada; 14 University of Calgary, Alberta Children’s Hospital, Calgary, AB Canada; 15 Department of Paediatrics, Western University, London, ON Canada; 16 Queen’s University, Kingston General Hospital, Kingston, ON Canada

**Keywords:** Experiences with care, Inherited metabolic diseases, Family support, Coping, Qualitative research

## Abstract

**Background:**

We sought to understand the experiences of parents/caregivers of children with inherited metabolic diseases (IMD) in order to inform strategies for supporting patients and their families. We investigated their experiences regarding the management of disease, its impact on child and family life, and interactions with the health care system.

**Methods:**

From four Canadian centres, we conducted semi-structured telephone interviews with parents/caregivers of children with an IMD who were born between 2006 and 2015 and who were participating in a larger cohort study. Participants were selected with the aim of achieving a diverse sample with respect to treatment centre, IMD, and age of the child. Interviews emphasized the impacts of the disease and its treatment on the child and family and explicitly queried perceptions of interactions with the health care system. We identified emergent themes from the interview data.

**Results:**

We completed interviews with 21 parents/caregivers. The 21 children were aged <1 to 7 years old with IMD that included amino acid disorders, urea cycle disorders, fatty acid oxidation disorders, and organic acid disorders or ‘other’ IMD. Most parents reported that they and their families had adapted well to their child’s diagnosis. Parents used proactive coping strategies to integrate complex disease management protocols into routine family life. An important source of stress was concern about the social challenges faced by their children. Participants reported positive interactions with their most involved health care providers within the metabolic clinic. However, they reported challenges associated with the health care system outside of disease-specific metabolic care, when encountering systems and providers unfamiliar with the child’s disease.

**Conclusions:**

The successful use of proactive coping strategies among parents of children with IMD in this study suggests the potential value of promoting positive coping and is an important direction for future study. Parents’ social concerns for their children were important stressors that warrant consideration by health care providers positioned to support families. Our results with respect to experiences with care highlight the important role of specialized metabolic clinics and point to a need for better coordination of the care that takes place outside the disease-specific management of IMD.

## Background

Inherited metabolic diseases (IMD) are a large group of individually rare single gene disorders, most often diagnosed early in life, that involve defects in metabolic pathways. They are characterized by a range of clinical manifestations that can include episodes of acute metabolic crises, insidious manifestations, or chronic multi-systemic sequelae [1]. Advancements in medical interventions have increased life and health expectancy in individuals with IMD, with new therapies appearing at an accelerating pace [2]. However, both disease manifestations and treatment regimens, which frequently involve highly specialized diets and medications, may be important sources of stress and have important impacts on quality of child and family life [3–7] and on the health care system [8].

Research to describe families’ perspectives and experiences in caring for children with IMD, both at home and when receiving care, can help clinical- and system-level providers to better understand how to optimize their support of children with IMD and their caregivers. In this qualitative interview study, we aimed to understand parents' experiences with the management of their child's IMD, the impact of the disease and its management on child and family life, and their perceptions about their interactions with the health care system.

## Methods

### Participants

This study was embedded in a larger, multi-faceted cohort study as part of the Canadian Inherited Metabolic Diseases Research Network (CIMDRN) [9]. CIMDRN is a pan-Canadian multi-disciplinary network that has established a consent-based cohort of >550 children, born between 2006 through 2015, diagnosed with one of 31 IMD (Table 1), and receiving care at one of 13 participating pediatric metabolic clinics. Eligible participants for the present study were parents/guardians of children enrolled in CIMDRN’s cohort study at one of four of these participating metabolic clinics (in Halifax, Montreal, Ottawa, and Vancouver, Canada) who had consented to be contacted to participate in an interview and/or survey. The selected participants were nominated by their families as the most knowledgeable of the child's disease and care.

From the CIMDRN cohort at the four centres listed above, for the present study we used a purposive sampling method to achieve a sample of parents/guardians that represented a diverse group of children receiving care at different centres, with different IMD, and of different ages [10]. The eventual sample size was dependent on data saturation [11]. Based on previous experience and published guidance [12, 13], we anticipated approximately 15-25 interviews. Selected eligible families were invited by telephone or email. Participants provided written informed consent prior to the interview. A maximum of three attempts were made to contact families, a voice message being left with each contact where possible. If no response was forthcoming or the family declined participation, no further contact was made.

### Data collection

Telephone interviews were conducted by a single researcher (SSi) using a semi-structured interview guide. The interview guide was informed by a broad scoping review of the literature [14] and revised through discussion among investigators with expertise in qualitative methods and clinical experience in treating IMD. Topics covered by the guide included well-being (mental, physical and social health) of the child and family, experiences with daily disease management at home, and interactions with the health care system, including diagnostic care and care associated with on-going disease management. A pre-test of the interview guide was conducted with a parent of a child with an IMD who was an advisor to the study team; data from the pre-test interview were not included in the analysis for the final study. All interviews were audio-recorded with participants’ consent and subsequently transcribed.

### Analysis

Seven members of the research team collaborated on an initial thematic analysis of the first three interview transcripts [15, 16], to identify broad categories and codes and to revise the interview guide. One researcher (SSi) coded all subsequent interviews, with review and verification by a second researcher (BKP). Analysis took place concurrently with the interview process so that emergent themes could be incorporated into subsequent interviews [17]. Discussions among team members were held regularly to review the transcripts, discuss emerging themes, assess the degree of saturation, and identify priorities for participant recruitment. We actively sought negative examples during the analysis of the data, i.e., findings that were inconsistent with the overall results that were emerging from the study. This was made possible through querying emerging themes in subsequent interviews. The analysis was supported by NVivo software (http://www.qsrinternational.com). Participants who consented to be re-contacted for this purpose received a preliminary summary of the study findings and were invited to submit any questions or comments to the investigators.

Ethics approval for this study was received from the Children's Hospital of Eastern Ontario Research Ethics Board, the Ottawa Health Science Network Research Ethics Board, the University of British Columbia/Children's and Women's Health Centre of British Columbia Research Ethics Board, and the McGill University Health Centre Research Ethics Board.

## Results

From 36 recruitment invitations, 21 caregivers provided signed informed consent and completed an interview. Even though data saturation with respect to main themes was judged to have been achieved with 13 interviews, we continued to interview all participants who had provided consent, to incorporate their views in recognition of their interest in the study, in order to enrich the data as much as possible across the diverse set of diseases involved, and because of the limited literature on family experiences with IMD.

Most participants (*n *= 18) were mothers of the child with an IMD (the other three participants were fathers or grandparents). Twelve different diseases were represented (Table 1); for the purposes of reporting and to protect the identity of families with these rare conditions, we categorized these as amino acid disorders, urea cycle disorders, fatty acid oxidation disorders, and organic acid disorders or ‘other’ IMD, when labelling participant quotations. The age of the children whose parents were interviewed ranged from under 1 year to 7 years. The vast majority of the children of participants (*n* = 17) were diagnosed with an IMD very early in life, through newborn screening programs. The average interview length was 55 min. Of participants who received a preliminary summary of the findings (*n* = 18), none provided comments or questions.

**Table 1:** Eligible IMD (12 of these 31 diseases were represented in this interview study, shown in italics, with multiple interviews for some diseases)

Amino acid disorders:
*Phenylalanine hydroxylase deficiency*
Homocystinuria
*Maple syrup urine disease*
*Tyrosinemia Type I*
Urea cycle disorders:
Arginase deficiency
Argininosuccinic acidemia
Carbamyl phosphate synthetase deficiency
*Citrin deficiency*
Citrullinemia
Hyperornithinemia-Hyperammonemia-Homocitrullinuria syndrome
N-acetylglutamate synthetase deficiency
*Ornithine transcarbamylase deficiency*
Fatty acid oxidation disorders:
*Medium chain acyl-CoA dehydrogenase deficiency*
Very long-chain acyl-CoA dehydrogenase deficiency
*Carnitine uptake defect*
*Long-chain 3-hydroxyacyl-CoA dehydrogenase deficiency*
Trifunctional protein deficiency
Organic acid disorders or ‘other’ IMD:
ß-Ketothiolase deficiency
*Glutaric acidemia type I*
HMG-CoA lyase Deficiency
Isovaleric acidemia
3-Methylcrotonyl-CoA carboxylase deficiency
Methylmalonic acidemias
Propionic acidemia
Guanidinoacetate methyltransferase deficiency
*Mucopolysaccharidosis type I*
Farber disease
*Galactosemia*
*Glycogen storage disease type 1*
Multiple carboxylase/biotinidase deficiency
Pyridoxine-dependent epilepsy

Participants’ descriptions of their children’s symptoms and management protocols were heterogeneous. Despite this, we identified key themes related to families’ experiences that were strikingly cross-cutting. We report the key emergent themes within the two broad topics on which the interviews focused: experiences with disease and its management at home and in the community and experiences with the health care system.

### Experiences with disease and its management at home and in the community

We organized the results under three key themes: (i) the ‘new normal’ and proactive coping; (ii) social stressors; and (iii) parent advocacy.

(i) The ‘new normal’ and proactive coping

The daily management of their child's IMD was described by many caregivers as intense, due to the need for the child (and sometimes the entire family) to learn to adhere to very restricted diets and treatment regimens. For example, many parents expressed difficulties associated with the early stages of adjusting to disease management:

*"…it was a really big adjustment for our family just learning new ways, like new ways of eating I guess, because we were not, we're not vegetarians*." (participant 11, amino acid disorder)

*"I would say the worst of it was when he was a newborn because they told us that we had to feed him exactly every 3 h."* (participant 18, fatty acid oxidation disorder)

Some parents took a leave of absence or even sacrificed their careers to learn and adapt to the complexities of the disease:

*"I never went back to work. I was a [occupation] and made more money than my husband. I gave up my job to take care of [child]...."* (participant 10, organic acid disorder or other IMD)

*"I took an extra year off just to make sure that I understood her diet properly and to make sure that I found a daycare that's going to be convenient."* (participant 11, amino acid disorder)

However, participants often reported that they eventually adjusted well to the complex day-to-day management, so that it became the 'new normal' for their family:

*"We don't know anything else… it keeps the family on a very strict routine because we know we have to get that breakfast, lunch and dinner into them. So, it's a very timetabled thing."* (participant 04, amino acid disorder)

*"But we have no choice…they're going to have to deal with that all their life and…they don't know any better…some people tell us we're lucky because we haven't had a kid that doesn't have it, so we don't know how it is like, how easy it would be if they wouldn't have [disease]."* (participant 17, amino acid disorder)

Indeed, some participants expressed that it was challenging to consider how the disease impacted their family life, since it was now all they knew:

*"Nowadays it's one of my routines. I know what he has…it's very simple for me right now, I know how to calculate and everything… We're so used to it you know?"* (participant 16, amino acid disorder)

*"We live in a new version of normal that is a heightened level of stress so… we live in the state of stress, it's just our new normal so I don't know that we notice it as much. It doesn't go away so it's just always there right?"* (participant 15, amino acid disorder)

This adjustment to life with an IMD seemed to be accomplished through the use of a range of coping strategies. Specifically, respondents provided numerous examples of the organization, planning, and lifestyle changes they had undertaken in order to cope with disease management and establish routines. For example, for one family this included a choice to live close to the child's school:

“*We are living in apartment in [area] and just a few steps from school…very close to the school just in case anything happened we can go to the school.” *(participant 06, organic acid disorder or other IMD)

For other families, it included selectively placing the child in a specific supportive day care or school:

*"I drive 20 min of out my way…of where I work and live just so I can take them there [child's daycare]. I think, you know, I trust them so, you know and they're really good with the kids and it's a family-run daycare…"* (participant 07, fatty oxidation disorder)

Planning ahead and being prepared for meals was described as important by several participants, for example:

*"We do basically 2 trips to the grocery store every week, and everything is planned on a 7 day calendar…Friday, Saturday I'll sit down and I'll plan out the meals for the week."* (participant 21, fatty acid oxidation disorder)

Organizing support from extended family members was another strategy mentioned by several participants:

*"…both our moms have the scale at their house, they have food for her. And then, we are hoping to go [State] in Spring break so my mom is gonna come along just to help us out."* (participant 03, amino acid disorder)

*"Our social life is pretty good because now we can drop him off at my in-laws, or my parents and we can go out, we can have a life."* (participant 10, organic acid disorder or other IMD)

Parents also used social media and the internet for accessing information and resources and for connecting with other families:

*"…the only thing is the support group on Facebook so if I have questions or whatnot it… it is nice to be able to go on there and get experiences from other parents."* (participant 11, amino acid disorder)

*"…for the first little while it was mainly just researching it on the internet…"* (participant 09, fatty acid oxidation disorder)

For many families, vacations were described as requiring a lot of planning and preorganization:

*"…you kind of have to visualize what you're going to do…what you need to pack because it's not just packing your clothes, we have to pack all the food, we have to bring their bread we have to… So that's a challenge."* (participant 17, amino acid disorder)

Another respondent described developing a spreadsheet to manage health care appointments:

*"I created a spreadsheet of all of our specialists, all the tests we have to do, so it's, you know it's an ongoing spreadsheet that I update… every 3, 4 months and the nice thing about it is that when I do update it…which is a lot of work but when I…do it then I know what I'm missing."* (participant 10, organic acid disorder or other IMD)

(ii) Social stressors

Despite these coping strategies and the sense that many families were coping well, participants often reported feeling stress related to concerns about the social lives of their children, particularly surrounding situations where the child may be excluded because of necessary dietary restrictions. These challenges intensified during holidays and special events:

*"…just going to children's birthday parties or things like that, it is getting a bit more difficult… she sees them eating this food, and then she'll look at hers and she knows that it's not the same right? Yeah so it is very, it is difficult…"* (participant 11, amino acid disorder)

Some parents/guardians described experiencing difficulties accessing special medical foods that are similar to foods eaten by unaffected children. Being able to identify with other children in this way was depicted as important for the social development of the child:

*“…but normality, being able to find something comparable to what all the other kids are eating, that's been a huge pain…She gets a little sulky sometimes, because she knows she is not getting exactly the same thing the kids get but we go out of our way to find kind of like the replica…”* (participant 01, organic acid disorder or other IMD)

Feeling isolated or excluded was identified by some parents as a particular worry for their child while at school:

*“… when the food comes in the teachers tend to give them a pencil or an eraser and we have now a bucket-full of pencils and erasers and that is a measurement of how often that they are left out of these things…”* (participant 04, amino acid disorder)

(iii) Parental advocacy

Perhaps in part as a response to social challenges, a third theme, parental advocacy, emerged when considering children's access to special services and inclusion in activities, particularly in the context of managing the diet, which required parental vigilance in order to prevent a child’s exposure to restricted foods:

*"…we just have to really advocate for her. We are part of a church, and our church in the infant nursery like would provide [brand name] crackers for kids and like she can't have [brand name] crackers and I didn't want them to handing out food so just kind of suggesting that they change the way they do things."* (participant 03, amino acid disorder)

*"We have* *[activity club for children]* *and the reason why I became a Unit Leader was because I needed to push the food out of weekly meetings because it was something, again, [child] would be exposed to it during the day."* (participant 04, amino acid disorder)

Some caregivers also described advocating on a broader level, with the government, in order to access needed foods or services:

*"…the only complaint I have about that is we…so we lobbied the [Provincial government]… we met with our [political representative] and we petitioned for coverage of low protein foods…."* (participant 15, amino acid disorder)

The rarity of IMD was sometimes perceived to necessitate parental advocacy in school settings:

"*…because the school board has never dealt with a child that has [disease]…I went in there with an 8 page letter…I kept telling the school board, if my son doesn't get the amount of hours that I requested, he will not go to your school."* (participant 10, organic acid disorder or other IMD).

### Experiences with the health care system

The two key themes that emerged within this broad topic were: (i) experiences with IMD-specific care; and (ii) experiences with non IMD-specific care.

(i) Experiences with IMD-specific care

Parents commonly reported that children required frequent health care interactions involving multiple providers from various disciplines. Almost all of the children in this study received disease-specific care from specialist metabolic physicians and dietitians. Most participants were highly satisfied with the care that their child received in the specialist metabolic clinic, with metabolic health care providers regarded as supportive, and dietitians in particular described as highly engaged with families:

*“…when we finally met [metabolic physician] … he gave us the time. At no time did we ever feel that we… needed to hurry up and get out and we were extremely happy.”* (participant 13, fatty acid oxidation disorder)

*“…the dietitians really help, like really help a lot, you know, um, really guiding me and everything with his intake with food and you know, trying new things.”* (participant 08, amino acid disorder)

*“… there was one person that changed our life throughout the whole process and that was [name of dietitian]…She was life-changing. Whoever is in her position is life-changing for someone in our position. She was incredible because she spoke to us in a way that we didn’t feel like we were going to end the world with certain decisions… she very much helped to relax us a little bit and to feel normal, like helping us to cope with our new normal.”* (participant 05, urea cycle disorder)

Respondents also commonly expressed satisfaction with the coordination of care in this part of the health care system:

*"Oh, when she was diagnosed it was great because [metabolic physician] and [dietitian] would come in together and we got a chance to see both. We have an option to see them separately. So yah, there is definitely a good coordination between them."* (participant 01, organic acid disorder or other IMD)

*"I find the [metabolic clinic] to be really well, really effective, really coordinated and great systems in [children's hospital] so that part has been really great."* (participant 15, amino acid disorder)

(ii) Experiences with non IMD-specific care

Although respondents expressed contentment with IMD-specific care, many participants reported negative experiences and dissatisfaction with non IMD-specific components of the health care system that were used frequently by their child, for example, the emergency department, the pharmacy, or the blood laboratory; and with coordination among these components of care.

For many children, frequent blood draws were required and were described as highly stressful due to the inherent pain:

*“There was a time we took [child] for a blood draw and I could hear her, and I could hear her screaming and [technician] was fishing in [child]'s arm for the vein. Like, excessively fishing and I asked her to stop. Blood draws were very, very difficult."* (participant 05, urea cycle disorder).

Some parents expressed their concerns about the specific services in phlebotomy, including access to highly experienced professionals, late results, or inconvenient hours:

*"…my son is very small…They have to take a lot of blood. And the people, I don't think they have enough experience to poke a child so young....there's another team for the just for the young…But we cannot access to this team."* (participant 14, urea cycle disorder)

*"…we do it at home with the finger. I receive the blood work of my son not on the second day, not on the third, later, so I don't know what's going on like, this week, my son could have the level very high for 1 week and I'm not gonna know, and the next week I'm going to receive the results."* (participant 12, amino acid disorder).

*"…having a clinic open Saturday mornings for even just one hour because we do blood work so regularly… it impacts a lot of people's schedules… eat up a lot of our vacation time, sick leave, parental leave, whatever we have… just for the blood work alone."* (participant 04, amino acid disorder).

Respondents also discussed challenges related to the internal coordination of the hospital. For example, this included concerns with receiving the correct medication from the pharmacy and access to special medical foods when the child was a hospital inpatient:

*"…we were told not to use medication from home …but the pharmacy, when she was in emerg that afternoon, they were going to send up her meds from pharmacy, which they didn't…So we gave her the meds that we already had, in which case they said – Don’t use the meds you have from home. Well, we didn’t hear from pharmacy again until the next morning."* (participant 05, urea cycle disorder)

*"…but they didn't have any soy based formula at all. And we have since had her in the hospital as she got older but we don't let her eat anything from the hospital because there is always something in it that she can't eat."* (participant 01, organic acid disorder or other IMD).

However, there were exceptions, with a small number of participants reporting positive experiences with the internal coordination of hospital departments. For example, one participant reported that their child’s special dietary needs were communicated well upon hospital admission and noted that the family appreciated the opportunity to bring and refrigerate foods from home:

*“Yeah we’ve never had an issue and in most cases the floor knows we’re coming up…and so the low fat menu is already in our room…. it’s been very smooth, I mean we all… once we’re admitted whoever goes home to shower or change or grab extra things…brings back some of her familiar snacks and there’s always a fridge in the room so we fill it…”* (participant 21, fatty acid oxidation disorder)

A second participant who was satisfied with care coordination attributed this in part to the family’s personal efforts to become involved in hospital activities and show their appreciation; and in part to the metabolic physician’s role in coordinating with other departments:

*“…We have had a need to kind of cross over into other departments…and we’ve found them to be all very supportive and helpful … part of it though is a bit self-created …we really try and reach out to them…when we walk into the hospital…everybody is excited to see [child], everyone knows who [child] is…we have great things to say but we work hard to make sure those relationships are there”* (participant 15, amino acid disorder)

*“…Our [metabolic physician] is really good at…making sure she’s up to date…on what those results from the other departments were and.. she has good relationships within the hospital.. again but it’s a person-dependent scenario…if you have a doctor who … nobody likes or if you have a doctor that is shy or not well connected, well then maybe my experience would be different….”* (participant 15, amino acid disorder)

Concerns about the care their child received in the emergency department was noted by several participants. Respondents commonly expressed their distress and dissatisfaction with the inconsistency in care received from the emergency department during a crisis:

*"… I just have a lot of stress around mostly around the health care that we received there [emergency department] because I can't say that it's consistent at all…I feel like almost like I have to prove that they are sick…"* (participant 02, fatty acid oxidation disorder)

This sometimes extended to worry about the capability of emergency professionals to manage the needs of patients with a rare (and unfamiliar to the provider) condition:

*"When we go to the ER in crisis…we know what to say, we know what's going to work… we've come up against a couple of doctors in the ER that don't appreciate that, they want to be the ones to… when they walk into the room, everyone looks to them for the answers."* (participant 21, fatty acid oxidation disorder)

In contrast, a small number of participants described having positive experiences and expressed satisfaction with the care their child received at the emergency department. In one case, this was attributed to a letter provided to the parents by the metabolic specialist clinic, to inform emergency providers about the illness:

*“…so we just took him right to emergency, and what helped is that they gave us a letter that said that he can have a metabolic crisis…so as soon as I checked in to the pediatric emergency, I gave them the letter and explained that it’s… you know… quite serious…And they got us in right away.”* (participant 18, fatty acid oxidation disorder)

However, another participant described an experience whereby the emergency letter was not taken seriously by a provider:

*“…the first time I had to bring* *[child]* *in with the protocol letter, the guy who’s a male nurse … he looked at it and he says, ‘What’s this?’ And I said, ‘It’s a protocol letter. I was told to give you that before I even handed over the (insurance card)’…He goes, ‘Well who is this… this doctor?’…and he was making fun of it there and he tossed the paper.”* (participant 13, fatty acid oxidation disorder)

In summary, participants often felt comfortable with the management of the major clinical manifestations of their child’s condition but expressed frustration with frequently used services outside of the specialist clinic. While these frustrations were often relayed as single anecdotal experiences, parents emphasized their importance as sources of stress that impacted quality of life. As one parent noted:

*“…quite often it’s very, very small things that make a massive difference in overall patient care.”* (participant 05, urea cycle disorder)

## Discussion

Parents of children with IMD in this study reported that they became well-adjusted to the complex daily management requirements of their child's disease and redefined their ‘new normal’ through the use of proactive coping strategies. Despite these coping strategies, many respondents reported stress related to concerns about the social lives of their children that necessitated parent advocacy, particularly surrounding situations where the child may be excluded because of dietary restrictions. Most parents interviewed were highly satisfied with care received in the specialist metabolic clinic. However, they often reported negative experiences with the non IMD-specific components of the health care system that their children used frequently. While these were often described to the interviewer as stories about single events, they were recounted across many interviews and parents reported these experiences as an important source of stress.

Our findings with respect to coping and the importance of social support and social challenges are supported by the small body of literature related to family experiences among children with an IMD. Parents of children with an IMD have been reported in other studies to adjust well to their child’s diagnosis [18–20], although the impact of IMD on parent and family well-being may depend on the specific disease and its manifestations [21]. Proactive coping or future-oriented thinking includes a set of strategies that involve anticipating probable risks and stressors that may cause harm and taking actions beforehand to prevent or reduce their impact [22, 23]. Previous research in this area has identified parents’ use of a range of strategies that included proactive coping, other positive coping strategies (e.g., religion, emotional/social support), and less adaptive means of coping (such as substance use) [24]. Similar to our study, others have identified social support, including close family and friend relationships [5, 20, 25, 26] and social media [27], as important to caregiver and family well-being. These informal support systems were commonly identified as important in our study, while the receipt of formal psychological or counselling services was mentioned less frequently by participants. To the extent that positive coping strategies may be associated with higher quality of life for the child and family, coping is a promising area for further research and potential supportive interventions.

Families’ concerns about the social development of children with IMD may be exacerbated during life transitions, particularly adolescence [25, 27], and may be more prevalent for IMD characterized by significant physical disability [25]. In our study we identified social challenges in families of relatively young children (up to age 7) that were mainly connected to the need for specialized diets, and thus we were unable to corroborate previous research about transitions to adolescence and adulthood. However, the persistence of these concerns about social development in studies of pediatric IMD strongly suggests a need to address children’s social needs alongside social support for parents. Indeed, recent qualitative studies found that for children with mucopolysaccharidoses and their families, concerns about ‘fitting in’ with peers and within broader society were dominant themes [28, 29]. Parent advocacy was an important theme that emerged in our study in part in relation to the social challenges described above and also extended to advocacy around access to care. We did not identify advocacy as a theme in related IMD literature. While we did not collect socioeconomic data for our study sample, the parents we interviewed may have been relatively socioeconomically advantaged, which may have afforded them greater resources and capacity to participate in advocacy activities. Supporting this possibility, a common parent-reported theme in the literature but not in our study is financial stress [6, 7, 21, 30, 31].

To our knowledge, ours is the first study to specifically describe differences in families’ experiences with disease-specific and non-disease-specific health care services for IMD. Previous studies have identified both positive and negative care experiences in this population [7, 31] and one previous study specifically identified challenges with care from providers unfamiliar with a child’s conditions (e.g., in the emergency department) [7]. Guidance for providing care for children with special needs suggests some potential strategies for addressing these challenges – for example, co-developed care plans, receipt of care within a ‘Medical Home’, relational continuity with a single key provider, facilitating collaboration across providers, and increasing family involvement as full partners in the care of their child, including health care decision-making [32–36]. Further research is needed to identify which specific strategies may best address the needs of children with IMD. Such research will be critical to help IMD centres develop effective approaches to communication with providers and systems both within and outside the metabolic clinic, for example, in order to: facilitate appropriate emergency management of metabolic crises in children with IMD; ensure that both health care providers and school-based professionals are aware of and support the special dietary and medication needs associated with IMD management; and identify approaches to improve the experiences of families who are frequent users of services such as phlebotomy.

Important strengths of this study include its wide representation of IMD and its in-depth insights generated about families’ experiences, both with home management and with receipt of health care. A further strength was our use of several strategies to ensure the rigour of the methods and verify the findings to enhance their credibility [37]. These strategies included participation of multiple investigators in reviewing the interview data, analyzing the data in parallel with the conduct of the interviews so that emerging results could be investigated in later interviews, and actively seeking and discussing negative examples i.e., findings that that were inconsistent with the overall emerging results, during the analysis.

A limitation of our study is that the small number of families with experience of any single IMD, or any single set of disease manifestations or treatment protocols, precluded us from fully investigating differences across disease characteristics. Previous research has identified differences in quality of life among parents of children with different IMD [21]. As well, other studies have identified diagnostic delays as a source of stress for families with IMD. Since the vast majority of children of our study participants were diagnosed through newborn screening, we had insufficient data to reflect upon this particular challenge, which deserves further study in future research involving children with rare diseases not identified within the first few weeks of life [30]. Furthermore, although we completed interviews with 58% (21/36) of families who were invited to participate in the study, those who did not participate may have been experiencing more challenges in coping, and thus were unable to devote time to participating in an hour-long telephone interview. Our findings thus warrant additional investigation in samples that are representative of the full population. Understanding the perspectives of families experiencing the highest intensity of stress, with respect to both needs and availability of resources, will be particularly challenging. This may require less burdensome methods of data collection to facilitate participation in research, for example, short surveys or brief in-clinic interviews. Such studies could also strive to incorporate validated measures of the psychosocial factors we identified as priorities, for example, coping and social support, to further elucidate families’ experiences and to investigate their association with quality of life. Future studies could also seek to better understand fathers’ experiences, given that most participants in our study were mothers. Finally, there is a need for research that emphasizes children’s own perspectives, particularly through adolescence and the transition to adult health care systems.

## Conclusion

The successful use of a range of proactive coping strategies among parents of children with IMD in this study suggests the potential value of promoting their use and is an important direction for future study. Parents’ social concerns for their children were important stressors that warrant consideration by health care providers positioned to support families. Our results with respect to experiences with care point to a need to better coordinate care across the entire health care system, including those components that are not specific to the management of IMD.

## Abbreviations

CIMDRN: The Canadian Inherited Metabolic Diseases Research Network
IMD: Inherited metabolic diseases
